# Chinese industrial air pollution emissions based on the continuous emission monitoring systems network

**DOI:** 10.1038/s41597-023-02054-w

**Published:** 2023-03-22

**Authors:** Ling Tang, Min Jia, Junai Yang, Ling Li, Xin Bo, Zhifu Mi

**Affiliations:** 1 School of Economics and Management, Beihang University, Beijing, 100191 China; 2 School of Economics and Management, University of Chinese Academy of Sciences, Beijing, 100190 China; 3 International School of Economics and Management, Capital University of Economics and Business, Beijing, 100070 China; 4 Department of Environmental Science and Engineering, Beijing University of Chemical Technology, Beijing, 100029 China; 5 BUCT Institute for Carbon-neutrality of Chinese Industries, Beijing, 100029 China; 6 The Bartlett School of Sustainable Construction, University College London, London, WC1E 7HB UK

**Keywords:** Sustainability, Industry

## Abstract

As the world’s largest industrial producer, China has generated large amount of industrial atmospheric pollution, particularly for particulate matter (PM), SO_2_ and NO_x_ emissions. A nationwide, time-varying, and up-to-date air pollutant emission inventory by industrial sources has great significance to understanding industrial emission characteristics. Here, we present a nationwide database of industrial emissions named Chinese Industrial Emissions Database (CIED), using the real smokestack concentrations from China’s continuous emission monitoring systems (CEMS) network during 2015–2018 to enhance the estimation accuracy. This hourly, source-level CEMS data enables us to directly estimate industrial emission factors and absolute emissions, avoiding the use of many assumptions and indirect parameters that are common in existing research. The uncertainty analysis of CIED database shows that the uncertainty ranges are quite small, within ±7.2% for emission factors and ±4.0% for emissions, indicating the reliability of our estimates. This dataset provides specific information on smokestack concentrations, emissions factors, activity data and absolute emissions for China’s industrial emission sources, which can offer insights into associated scientific studies and future policymaking.

## Background & Summary

China has been suffering from severe air pollution^1^. Industrial sectors contributed the majority of China’s air pollutant emissions, representing 72.8–86.1%, 74.3–91.0% and 40.7–79.1% of national anthropogenic particulate matter (PM, comprising all PM particle sizes)^1,2^, sulphur dioxide (SO_2_)^1–3^ and nitrogen oxide (NO_X_)^1–3^ emissions, respectively, between 2010 and 2018. These air pollutants constituted the primary precursors of PM_2.5_ (PM with an aerodynamic diameter within 2.5 μm) pollution, which poses severe environmental problems and public health burden^1^.

To control the industrial emissions, a nationwide, dynamic and up-to-date emission inventory is critical for accurately analysing industrial emission characteristics and targeted policymaking. There are some bottom-up emission inventories of atmospheric pollutants for China’s industrial emissions, including the Multi-resolution Emission Inventory for China (MEIC)^1,4^, the Regional Emission inventory in ASia (REAS)^5^, the Community Emissions Data System (CEDS)^6–8^ and other emission datasets^9–14^. However, due to the lack of actual monitoring data, these databases resort to average emission factors (i.e., atmospheric pollutant emissions per unit of production or fossil fuel combustion) compiled by previous studies^15,16^ or official guidebooks^17,18^ (such as the National Pollution Source Census^17^ published by the Council of State Governments (CSG)), which bears several shortages. First, these average emission factors do not entail direct CEMS-monitored observations but are proxies for various assumptions or indirect parameters (about operational conditions and control measures), which result in high uncertainty^19^. Second, based on many indirect parameters and associated assumptions, the emissions factors employed in previous inventories are assumed invariable within a given province^12,14^, region^13^ or nation^1,4^, thereby failing to reflect individual heterogeneities throughout industrial facilities^20^. Third, available emissions factors have been evaluated up to 2017^21^, and the effect of latest mitigation policies^22,23^ on industrial sectors through technology upgrading and operational adjustment has not been considered. Therefore, introducing direct and real-time CEMS-monitored observations can significantly reducing the estimation uncertainty due to the application of indirect and constant average emission factors.

China has started building a national continuous emissions monitoring system (CEMS) network (http://www.envsc.cn/) for high-emitting industrial stationary sources (such as operating units, machines, facilities or boilers for production) since 2007^24^, to directly measure hourly and source-level PM, SO_2_ and NO_X_ concentrations^20,25–30^. During 2015–2018 period, the CEMS network involved comprehensive industrial sectors, particularly energy- and emission-intensive sectors such as thermal power industry (representing 57.7%–77.1% of plants and 95.9–97.4% of total national capacity), iron and steel industry (representing 62.9%–71.6% of plants and 74.2–88.3% of crude steel production) and cement industry (representing 63.5%–77.2% of plants and 78.9–87.6% of total clinker production). To improve the quality and reliability of CEMS system, China has implemented a number of policy actions: developing detailed specifications and technical guidelines for CEMS’ proper operation, preservation and regulation^27,31^; conducting quarterly random inspections to avoid data manipulation^32^; and comparing monitoring values among emission sources to determine outliers^33^. To date, some research has employed CEMS data to analyse industrial emissions from limited industries, including thermal power industry^20,26,34^, iron and steel industry^28^ and cement industry^35^. However, these data based on actual monitoring measurements have not yet been extended to other industrial sectors, and a comprehensive analysis across different industry sectors has not been performed.

Here, we contribute to addressing above research gap by developing a new nationwide database of industrial emissions based on CEMS measurements, named Chinese Industrial Emissions Database (CIED). The CIED database considers comprehensive industrial sectors in China from 2015 to 2018, adding up to 10,933 plants and 19,032 facilities. In particular, the database introduces all available actual monitoring data of smokestack concentrations from CEMS network (exclusively provided by the China’s Ministry of Ecology and Environment (MEE), http://www.envsc.cn/) for PM, SO_2_ and NO_X_ from industrial plant stacks across China during 2015–2018, and estimates nationwide, real-time and dynamic industrial emission factors and absolute emissions on a source and monthly basis. The CEMS data can sufficiently provide a direct, simple estimates of nationwide, source-level and dynamic emission factors and absolute emissions for Chinese industrial sectors, which can address the above three limitations of using average emission factors. First, the CEMS database offers real-time measurements that avoid using diverse assumptions and indirect parameters employed in average emission factors of previous emission inventories and thus reduce the estimation uncertainty^36^. Second, the hourly, source-level actual CEMS measurements enhance the spatio-temporal resolutions of emission factors and absolute emissions, which can effectively highlight the heterogeneous and dynamic characteristics of industrial emissions over periods^26,37^. Third, the CEMS-monitored observations for the 2015–2018 period are applied, and the detailed, up-to-date emission factors and emission inventory are updated, directly reflecting the potential emission reduction effects of recent air pollution control policies^22,23^. Moreover, the CIED dataset also encompasses other stack specific information derived from the MEE (http://permit.mee.gov.cn/), regarding geographic allocations (e.g., latitude and longitude), physical parameter (e.g., diameter, height and temperature) and so on. In addition, uncertainty analyses for total emissions of PM, SO_2_ and NO_X_ across 2015–2018 show that our estimates are more robust (with 95% confidence interval (CI) of [−0.2%, 0.1%]) relative to prior studies (with 95% CI of [−76.0%, 136.0%])^10,13,38–42^). This CEMS-based CIED dataset can be employed to conduct a more accurate analysis of overall, detailed and dynamic characteristics of industrial emissions, serve mitigation policy making for China, and offer insights for other countries looking to control industrial emissions^43,44^.

## Methods

### Scopes and databases

The CIED database encompasses comprehensive industrial sources in mainland China from 2015 to 2018 in all the provinces and municipalities (totaling 26 and 5, respectively) in mainland China. According to the Industrial Classification for National Economic Activities (GB/T 4754–2017)^45^, these industrial emission sources can be aggregated into 10 sectors or 33 subsectors (deposited at figshare^46^). Thereafter, these sectors can be further divided into 170 subcategories (by fuel types, processes or products; deposited at figshare^46^). Specifically, by fuel type, thermal power industry is classified into 5 subcategories (i.e., coal, gas, oil, biomass and other fuels-based burning thermal power industries), according to the varieties of fossil energies used in the power generation. By process, iron and steel industry is allocated to 7 production processes (i.e., sintering, pelletizing, coking, ironmaking, steelmaking via a basic oxygen furnace, steelmaking via an electric arc furnace and steel rolling) or 22 associated processes (e.g., sinter machine heads in sintering); and cement industry can fall into 2 processes of kiln heads and kiln tails. By product, other subsectors are classified into 141 subcategories.

The CIED dataset is a new dataset that offers nationwide, detailed, dynamic emission factors and total emissions of PM, SO_2_ and NO_X_ between 2015 and 2018 for Chinese industrial sources across different fuel types, production processes or products categories. Compared with existing inventories^1,4–14^, the CIED dataset has the unique advantage in reducing estimation uncertainty by using real CEMS-monitoring data rather than average emission factor (and many assumption and uncertain parameters thereof).

In particular, the CIED dataset incorporates two databases, i.e., the CEMS database and source-specific information. The CEMS database—actual, source-level and hourly monitoring data of smokestack concentrations of PM, SO_2_ and NO_X_ for stationary industrial emission sources—are recorded by Chinese CEMS network and released by the MEE. Overall, a total of 17,134 sources (associated with 7,708 generating plants) across different industrial sectors are encompassed in the CEMS network from 2015 to 2018.

Source-specific information is provided by national facility-level database for Chinese industrial sources. Specifically, the facility-specific information for each individual industrial source involves activity level (industrial production or power generation, inputs and fuel consumption; yearly), unit or facility property (geographical location, production processes involved, emission sources associated, facility type, facility scale and age), quality of fuel and raw material and pollution control technology (category and removal efficiency), which are derived from the MEE^20,29,35^.

### Pre-processing of CEMS data

The CEMS includes a sampling system (to filter and sample flue gases), an analysis system (to evaluate flue gas indicators, especially smokestack concentrations) and a data-processing system (to collect, process and report real-time measurements)^24,27^. These three systems of CEMS should be carefully operated, maintained and examined, in order to prevent observation biases mainly in the sampling and analysis systems (in terms of zero drift, span drift and indication errors) and invalid data communication and data loss in data-processing system (leading to null and invalid values)^27^.

To ensure the quality of CEMS data, Chinese governments have promulgated a series of measures to prevent systematic biases, including: formulating detailed specifications and technical requirements for local government agencies and industrial plants to perform and superintend the proper operation, maintenance and regulation of CEMS network^27,31^; performing quarterly random examinations to prevent data manipulation^32^; comparing data among emission sources to identify outliers^33^; mandating plants to regularly calibrate, maintain and verify CEMS instruments^24,47^; and employing third parties to conduct technical validation for the CEMS network^24^. According to these official documents, all state-monitored firms are required to post their CEMS measurements to the local authorities via associated provincial online platforms. Then, the local governments randomly check the authenticity of the reported data at least once per quarter^20,27,32^, and publicly disclose the relevant inspection results through the same online platforms^20,48,49^. The firms that engage in data manipulation (including deletion, distortion and falsification of CEMS data, etc.) are subject to strict financial and criminal penalties^50,51^.

Even with all the above efforts, there are still a small number of invalid values in the CEMS database (accounted for 2.6%–3.3% of the total data from 2015 to 2018, deposited at figshare^46^), including missing data (nulls), zeros and abnormal (or extreme) observations, which should be seriously handled in accordance with relevant official documents and guidelines issued by the Chinese government. In general, nulls or zeros can be treated in three different methods depending on the duration^24^. First, we treat the nulls or zeros that last below 5 day(s) as invalid values and set nulls or zeros successive from 1 to 24 hour(s) to the averages of the two closest valid values before and after them^24,52^:

1 $${\widehat{C}}_{f,i,y,m,h}=\frac{{C}_{f,i,y,m,h-p}+{C}_{f,i,y,m,h+j}}{2}$$

where *C*_*f, i, y, m, h*_ is the stack gas concentration (g m^−3^) monitored by CEMS network, which denotes the real-time measurements of pollutant *f* produced by facility *i* in year *y*, month *m* and hour *h*; ${\widehat{C}}_{f,i,y,m,h}$ represents the estimated value for the nulls or zeros *C*_*f, i, y, m, h*_; *C*_*f, i, y, m, h*-*p*_ and *C*_*f, i, y, m, h*+*j*_ indicate the closest last valid values (*p* hour(s) before) and next valid values (*j* hour(s) after), respectively, of the nulls or zeros *C*_*f, i, y, m, h*_. Second, the nulls or zeros consecutive for more than 24 hours but less than 5 days are interpolated with the effective monthly averages near the time^24^:

2 $${\widehat{C}}_{f,i,y,m,h}={\overline{C}}_{f,i,y,m,\bullet }$$

where ${\overline{C}}_{f,i,y,m,\bullet }$ means the average of the hourly known values for the same atmospheric pollutant, production facility, operating year and month as *C*_*f, i, y, m, h*_. Conversely, we consider nulls or zeros successive for more than 5 days as an overhaul and ignore them^24^, in the light of estimation regulation. Furthermore, we use a data visualization to identify extreme data (in terms of the values outside the CEMS instruments’ measurement range) as outliers and treat those data in a same way as nulls (or zeros) in accordance with the authoritative regulations^24^.

### Estimation of emission factors and emissions

Using actual CEMS-monitored observations for nationwide industrial sources, we can directly measure the emission factors for PM, SO_2_ and NO_X_ on a source and hourly basis, which is the main contribution of this work and can enhance the estimate accuracy and avoids the use of various assumptions or indirect parameters that are common in existing research^20,26,29,35^:

3 $$E{F}_{f,i,y,m,h}={C}_{f,i,y,m,h}{V}_{i,y}$$

where *EF*_*f, i, y .m, h*_ stands for the emission factor (g per activity data), expressed as the emissions mass per unit of production or fuel consumption; *V*_*i, y*_ denotes the theoretical flue gas rate (m^3^ per activity data), defined as the volume of flue gas per unit of product or fuel consumption. Given that the CEMS equipment installed at smokestacks are required to monitor the abated smokestack concentrations after the effect of pollution control technology (if any), the abated emission factors can be estimated in a direct way without considering the removal efficiency-related parameters^24^.

Since the clean air policies and relevant regulations mainly focus on emission concentrations, vast quantities of other monitoring observations (especially flue gas rates) are missing from the CEMS database. Therefore, the application of theoretical flue gas rates in our estimation can significantly prevent serious underestimation of the actual flue gas volume due to these missing data^20,26,29^ and flue gas leakage^26,53^. Such theoretical values are estimated according to the systematic field measurements and analogy method conducted by the CSG^17,21^ and MEE^54,55^, with values determining by detailed products, process, scale, raw material, technologies, and fuel types. Accordingly, the actual flue gas rate can be obtained by multiplying the theoretical flue gas rate with the real industrial production or fuel consumption. Furthermore, we examine the theoretical flue gas rates based on the actual flue gas volume from CEMS monitoring samples for thermal power industry, iron and steel industry and cement industry (covering 1516, 210 and 919 facilities, respectively). Our estimates indicate that the actual values of flue gas rates generally approach their corresponding theoretical ones, within the uncertainty range (defined as the lower and upper bounds of a 95% confidence interval around the central estimates^42^) of ±10.1%, ±12.1% and ±6.7% respectively, at the 95% confidence level (deposited at figshare^46^). The results are consistent with the finding of existing studies^20,26,29^ and confirm the application of theoretical flue gas rates.

Then, we estimate the total emissions of PM, SO_2_ and NO_X_ for Chinese industrial sectors by multiplying the emission factors by the activity data, on a source and monthly basis^19^:

4 $${E}_{f,i,y,m}=E{F}_{f,i,y,m}{A}_{i,y,m}$$

where *E*_*f, i, y, m*_ indicates the absolute atmospheric pollutant emissions (g); *A*_*i, y, m*_ means the activity level, defined as the total amount of production (e.g., kg for crude steel in iron and steel industry) or fossil fuel consumption (kg for solid or liquid fuels and m^3^ for gas fuels).

In the CIED dataset, we calculate the total emissions on a monthly scale, in which emission factors are aggregated from hourly values to monthly values. Notably, the comprehensive annual, facility-level activity data is only available for three industrial subsectors (i.e., thermal power industry^20,26^, iron and steel industry^29^ and cement industry^35^) for 2015–2018. Therefore, we need to use the production data on a monthly basis and a provincial scale as the weights to assign the yearly facility-level activity data^26^:

5 $${A}_{i,y,m}=\frac{{A}_{{p}_{i},{s}_{i},y,m}}{{\sum }_{m=1}^{12}{A}_{{p}_{i},{s}_{i},y,m}}{A}_{i,y}$$

where the subscript *s*_*i*_ indicates the industrial subcategory *s* to which facility *i* belongs; *p*_*i*_ means the province *p* where facility *i* is located; ${A}_{{p}_{i},{s}_{i},y,m}$ denotes the monthly provincial production of industrial subcategory *s* in province *p*, which is derived from the official statistical yearbook (i.e., *Chinese Energy Statistics* *Yearbooks*^56^ *and China Statistical* *Yearbooks*^57^) and reports (available at http://www.cementchina.net/). Given that the lack of comprehensive facility-level activity data for other 30 subsectors (covering 74 types of industrial products), we directly use monthly province-level activity data (derived from *China Statistical Yearbooks*^57^), i.e., ${A}_{i,y,m}={A}_{{p}_{i},{s}_{i},y,m}$, or scale annual data (from *China Statistical Yearbooks*^57^, *China Mineral Resources*^58^, *China’s Building Materials Industry Yearbook*^59^ and the association of China refractories industry) to monthly levels using the proxies of monthly production of counterpart products.

### Uncertainties

We consider the uncertainties stemming from the volatility in the CEMS-monitored observations, theoretical flue gas rates and estimated monthly activity data, assuming that uncertainties in these three parameters are independent. Using Monte Carlo approach, we perform the systematic uncertainty analyses to examine the reliability and robustness of our estimated results introducing actual CEMS measurements. The detailed analysis steps are as follows: (a) assume the probability distributions for each tested model variable (CEMS-based smokestack concentrations, theoretical flue gas rates or activity levels) and obtain the related distribution parameters (e.g., mean and the standard deviation) as inputs to the Monte Carlo simulation; (b) produce random values following their respective probability distributions through Monte Carlo approach; (c) input random values to Eqs. (3–5) to generate a new group of emission factors and absolute emissions; and (d) run steps (b) and (c) for 10,000 times to obtain the range of uncertainty in our estimations in terms of 2 standard deviations (s.d.) of the above 10,000 sets of results^19,42,60^. Our results indicate that the estimates are relatively stable, with 2 s.d. compared with the associated mean (in %; reflecting the uncertainty ranges of our estimates) within ±7.2% for emission factors and ±4.0% for absolute emissions (Table 1). In particular, based on the detailed source-level activity data, uncertainty ranges in our estimates for three subsectors (i.e., thermal power industry, iron and steel industry and cement industry) are relatively small (±6.8% for emission factors and ±0.2% for emissions), compared to that for other subsectors (±7.2% and ±4.8%, respectively).

**Table 1:** Percentage uncertainty of the emission factors and emissions in CIED.

Sectors	Variables contributing to uncertainties of emission factors			Variables contributing to uncertainties of emissions			
	CEMS data	Theoretical flow gas rates	Altogether	CEMS data	Theoretical flow gas rates	Activity data	Altogether
Mining and quarrying	±5.85%	±3.80%	±7.13%	±4.06%	±3.03%	±2.26%	±5.08%
Manufacture of foods and tobacco	±5.76%	±3.69%	±6.92%	±5.62%	±3.59%	±1.98%	±6.79%
Light industry	±5.80%	±3.83%	±7.10%	±4.26%	±3.02%	±1.46%	±5.40%
Processing of petroleum, coking and processing of nuclear fuel	±5.70%	±3.76%	±6.92%	±4.49%	±3.26%	±1.77%	±5.42%
Manufacture of chemical products	±5.82%	±3.77%	±7.03%	±3.49%	±2.90%	±1.98%	±4.69%
Manufacture of non-metallic mineral products	±5.77%	±3.79%	±7.07%	±3.00%	±2.94%	±1.46%	±3.74%
Smelting and pressing of ferrous metals	±0.23%	±0.99%	±1.02%	±0.19%	±0.09%	±0.25%	±0.31%
Smelting and pressing of nonferrous metals	±5.82%	±3.76%	±6.99%	±4.26%	±3.05%	±2.61%	±5.62%
Manufacture of equipment	±5.84%	±3.88%	±7.23%	±5.65%	±3.63%	±1.29%	±6.82%
Production and distribution of electric power	±3.63%	±5.57%	±6.75%	±0.30%	±0.21%	±0.16%	±0.37%
**Overall**	±5.84%	±5.57%	±7.23%	±3.23%	±2.50%	±0.89%	±3.99%

### Uncertainties in CEMS data

To examine the volatilities in the high frequency CEMS data, we assume probability distributions (in a uniform distribution) for source-specific and monthly concentrations of each atmospheric pollutant, according to the tolerance ranges issued by the official regulation (HJ/T75-2007)^24^. In detail, a set of legal CEMS measures are mandated to control the systematic errors within ±15%, ±5% and ±5% for PM, SO_2_ and NO_X_ concentrations, respectively. Regarding the emission sources without CEMS, we use bootstrap simulation to randomly select samples from facilities with CEMS that in the same region, over the same period, and of similar emission source, fuel type and production process. Then, a Monte Carlo method is employed to generate random samples of pollutant concentrations following the associated distributions, and the simulations are conducted for 10,000 times to calculate the uncertainty ranges for emissions factors and absolute emissions (in terms of 2 s.d.). Our estimates indicate that the uncertainties ranges for emission factors and total emissions are within ±5.8% and ±3.2%, respectively (Table 1).

### Uncertainties in theoretical flue gas rates

In our estimation, the introduction of theoretical flue gas rates might arise uncertainty due to the large amount of missing data on real-monitoring flue gas rates from CEMS networks. Although this approach has the advantage of preventing severe underestimation and flue gas leakage, uncertainties may be attributed to the regardless of heterogeneities among individual facilities in production technologies, operational conditions and feedstocks, etc. Under such background, we calculate the uncertainty ranges based on the CEMS-monitored samples for 1,373, 210 and 919 facilities of thermal power industry, iron and steel industry and cement industry, respectively; and perform a single-sample two-tailed *t*-test (deposited at figshare^46^) for each subcategory of these three industrial sectors. The results demonstrate that the actual CEMS monitoring flue gas rates mostly close to their theoretical values in our estimates, within a likely range of ±12.1% at a 95% confidence level. Then, the Monte Carlo technique is employed to generate random values of flue gas rates uniformly distributed in the relevant uncertainties ranges. In addition, we use the maximum ranges for the industrial sectors without uncertainty ranges (e.g., ±10.07% for thermal power facilities burning oil). With 10,000 simulations, our analysis indicate that uncertainty ranges, represented by 2 s.d., are quite small, within ±5.6% and ±2.5% for emission factors and emissions, respectively (Table 1).

### Uncertainties in activity levels

To explore the uncertainty generated in the allocation of facility- and province-level activity data from yearly to monthly, we set a normal distribution with a 5% coefficient of variation (CV, the standard deviation divided by mean) for three subsectors of thermal power industry, iron and steel industry and cement industry with comprehensive facility-specific activity level^20,26,29,35^, and 10% CV for other industrial sectors according to existing literature^61^. Besides, the Monte Carlo method is conducted to produce random monthly activity values for estimated each facility or province. Relying on a total of 10,000 simulations, the uncertainty range in terms of 2 s.d. of all simulation results for absolute emissions is only ±0.9% from 2015 to 2018 (Table 1).

## Data Records

The CIED datasets^46^ are available at 10.6084/m9.figshare.c.6269295. It is organized as a set of excel datasets according to indicator and date. The indicators including emission concentrations, emission factors, activity data, absolute emissions and additional descriptions (including subcategories description, flue gas rates, comparison of uncertainty, invalid data and CEMS ranges); the date covers four years from 2015 to 2018. After merging, there are 5 excel datasets with 22 sheets provided at Figshare. In particular, the CIED database provided the high-resolution data at a source and monthly basis.

**Fig. 1:**
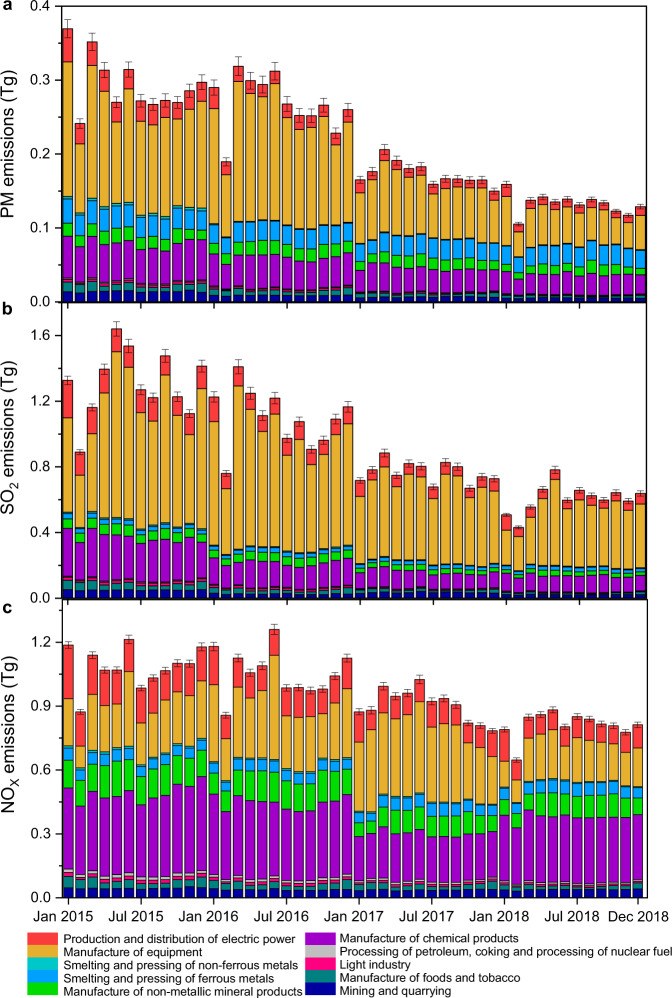
Estimated emissions of Chinese industrial sectors from 2015 to 2018. (**a**–**c**), Estimated monthly industrial emissions (Tg) of PM (**a**), SO_2_ (**b**) and NO_X_ (**c**). The error bars represent the uncertainty ranges.

The CIED dataset introduces actual systematic smokestack concentration measurements from China’s CEMS network and source-specific activity level from the MEE to directly estimate Chinese industrial emissions. In particular, the dataset presents systematic, dynamic, detailed emission factors and total emissions for PM, SO_2_ and NOx from China’s industrial sources during 2015–2018, by region (including 26 provinces and 4 municipalities) and sector (33 subsectors and 170 subcategories; Fig. 1).

## Technical Validation

### Independent verification

The estimates drawn from the CEMS data need careful verification against other independent data, which can also provide insight as to how the large emission reductions in industrial sectors (based on CEMS data) translate to trends in regional atmospheric concentrations. Therefore, we conduct an independent verification against an atmospheric dataset (i.e., ground-level measurements by air-quality monitoring stations). In particular, we compare the changes in industrial emissions from 2015 to 2018 (based on CEMS data) with those in regional atmospheric concentrations, both at the national level and in the top 10 provinces with the largest atmospheric emissions as of 2018. This experimental design was also used in existing studies^62,63^. In each of the 10 provinces, the industrial sectors has already been shown to have a large contribution to air pollution^64–71^. These provinces include Anhui, Guangdong, Jiangsu, Zhejiang, Shandong, Hubei, Chongqing, Henan, Hebei and Inner Mongolia (ranked by atmospheric emissions).

**Fig. 2:**
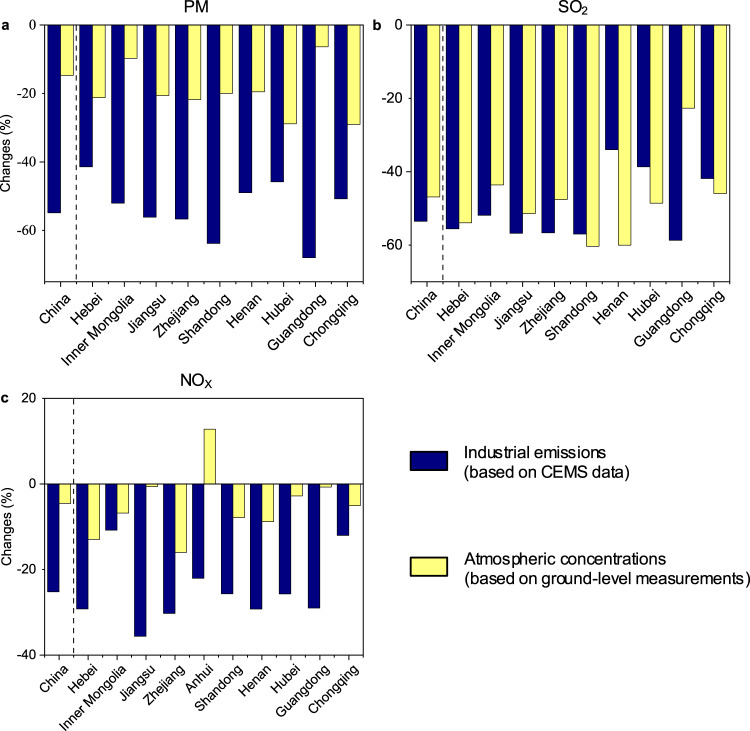
Independent verification against atmospheric concentrations. **a**–**c**, Changes in industrial emissions (blue bars) for PM (**a**), SO_2_ (**b**) and NOx (**c**) and the associated ambient concentrations from 2015 to 2018, at the national level and China’s top 10 provinces of the largest atmospheric emissions as of 2018. To verify the atmospheric impact of the emission changes for PM, SO_2_ and NO_X_ (based on the CEMS data), ground-level PM_10_, SO_2_ and NO_2_ concentration observations by national air-quality monitoring stations (yellow bars) are employed.

The ground-level PM_10_, SO_2_ and NO_2_ concentrations measured by national air-quality monitoring stations are employed to verify the atmospheric impact of the changes in PM, SO_2_ and NO_X_ emissions, respectively; these data are obtained from China National Environmental Monitoring Center (http://www.cnemc.cn/). The large reductions in PM, SO_2_ and NO_X_ emissions from China’s industrial sectors largely correlated with ground-level monitoring data. As shown in Fig. 2, the changes in all PM, SO_2_ and NO_X_ associated atmospheric concentrations (yellow bars of Fig. 2) are generally similar to the changes in emissions from industrial sectors (blue bars).

### Comparisons with existing emission databases

**Fig. 3:**
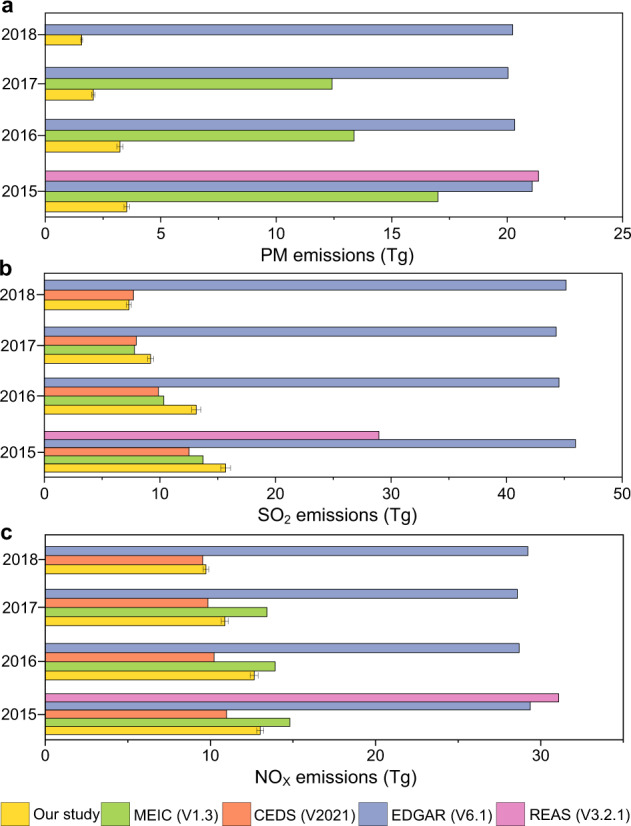
Comparison of estimated Chinese industrial emissions between 2015 and 2018. (**a**–**c**), The estimated industrial emissions in China (Tg) for PM (**a**), SO_2_ (**b**) and NO_X_ (**c**) in our dataset (yellow bars) and in existing datasets (MEIC (www.meicmodel.org); REAS (https://www.nies.go.jp/REAS/); CEDS (https://github.com/JGCRI/CEDS); the Emissions Database for Global Atmospheric Research (EDGAR) (https://edgar.jrc.ec.europa.eu/); non-yellow bars). The error bars denote the related uncertainties.

For verification, we compare our estimates for Chinese industrial emissions to previous datasets, as illustrated in Fig. 3. The results show that our estimates (based on the real measurements) are generally 85.15%, 20.32%, 23.21% below previous estimates. This is because existing studies resort to utilizing indirect average emission factors that were estimated up to 2017, overlooking the latest mitigation effects, especially associated with the upgraded pollution control technologies^5–9^ for PM, SO_2_ and NO_X_ respectively. In addition, the uncertainty analysis shows that our estimation exhibits a relatively low uncertainty level (with 95% CI of [−0.2%, 0.1%]) compared to existing studies (with 95% CI of [−76.0%, 136.0%]; deposited at figshare^46^)^10,13,38–42^, by using real, hourly and facility-level CEMS measurements.

## Usage Notes

The CIED dataset is subject to several limitations. First, China’s CEMS network has not yet covered all industrial emission sources, and these samples can be collected and incorporated to extend a complete CIED database in the future. Second, besides air pollutants, the CIED dataset can also introduces the real measurements of greenhouse gases (particularly CO_2_) and water pollutants, to support a comprehensive analyse of climate change policies and clean air policies for Chinese industrial emissions. Third, to enhance the accuracy of the estimation, future work can incorporate comprehensive high-frequency operational data (including activity data and flue gas rates) for each facility. Fourth, although Chinese governments have issued a range of stringent regulations^30^ to guarantee the reliability of the CEMS system, the careful verification, for example, comparing CEMS data with satellite data^27^ or ground-level monitoring data^28^, is valuable for verifying the results drawn from the CEMS data. Given that, we would update our database in the future if data are available.

## Data Availability

The CIED datasets are available in the form of XLSX files. No custom code is used in the construction of the datasets.
